# The Impact of Bushfire Smoke on Cattle—A Review

**DOI:** 10.3390/ani11030848

**Published:** 2021-03-17

**Authors:** Benjamin Eid, David Beggs, Peter Mansell

**Affiliations:** Melbourne Veterinary School, University of Melbourne, Melbourne, VIC 3010, Australia; eidben@hotmail.com (B.E.); pmansell@unimelb.edu.au (P.M.)

**Keywords:** bushfire, smoke, cattle, pollution

## Abstract

**Simple Summary:**

In 2019–2020, Australia had a particularly bad bushfire season which resulted in large numbers of people and animals being exposed to smoke haze for several weeks. We conducted a literature review to examine the evidence for effects of prolonged exposure to bushfire smoke on cattle. There was general agreement that small airborne particulate matter in smoke is the substance most likely to cause problems. There was indirect evidence about effects on cattle caused by other types of pollution containing particulate matter. We found little evidence to support severe effects on cattle. This may be because cattle do not tend to suffer from the co-morbidities that, in the human population, seem to be made worse by smoke and pollution. However, small changes to death rates or disease that is not severe may go unreported, so further study is warranted.

**Abstract:**

In 2019–2020, a particularly bad bushfire season in Australia resulted in cattle being exposed to prolonged periods of smoke haze and reduced air quality. Bushfire smoke contains many harmful pollutants, and impacts on regions far from the fire front, with smoke haze persisting for weeks. Particulate matter (PM) is one of the major components of bushfire smoke known to have a negative impact on human health. However, little has been reported about the potential effects that bushfire smoke has on cattle exposed to smoke haze for extended periods. We explored the current literature to investigate evidence for likely effects on cattle from prolonged exposure to smoke generated from bushfires in Australia. We conducted a search for papers related to the impacts of smoke on cattle. Initial searching returned no relevant articles through either CAB Direct or PubMed databases, whilst Google Scholar provided a small number of results. The search was then expanded to look at two sub-questions: the type of pollution that is found in bushfire smoke, and the reported effects of both humans and cattle being exposed to these types of pollutants. The primary mechanism for damage due to bushfire smoke is due to small airborne particulate matter (PM). Although evidence demonstrates that PM from bushfire smoke has a measurable impact on both human mortality and cardiorespiratory morbidities, there is little evidence regarding the impact of chronic bushfire smoke exposure in cattle. We hypothesize that cattle are not severely affected by chronic exposure to smoke haze, as evidenced by the lack of reports. This may be because cattle do not tend to suffer from the co-morbidities that, in the human population, seem to be made worse by smoke and pollution. Further, small changes to background mortality rates or transient morbidity may also go unreported.

## 1. Introduction

Australia has a long history of bushfire activity. The combination of an often hot and dry climate and large amounts of highly flammable native vegetation means bushfires are a regular occurrence in the Australian landscape [1]. The recent Australian bushfires of 2019–2020 were some of the worst bushfires seen in Australia’s history, and the worst ever recorded in the state of New South Wales (NSW) [2]. In NSW alone, 5.4 million hectares burned, and 800 million animals were killed as more than 11,000 fires burned over the course of months [2]. The smoke generated from the bushfires severely reduced air quality, with Australia’s capital city, Canberra, suffering through an air quality index more than 23 times the hazardous level, and the cities of Brisbane, Sydney and Melbourne also experiencing high levels of bushfire smoke for several weeks.

Bushfire smoke is known to contain many toxic pollutants [3], and many studies have investigated the detrimental effects on human health, often focused on effects to the respiratory system. High levels of particulate matter (PM) present in bushfire smoke appear to cause many of the health problems observed [4]. It is estimated that globally, landscape fire smoke is responsible for just under 340,000 human deaths per year [5]. Particulate matter is also a significant component of ambient air pollution, which in 2016 was responsible for approximately 4.2 million premature deaths worldwide [6].

Smoke from bushfires can travel hundreds of kilometres, leading to hazardous air quality in regions or cities far from the fire activity [7]. Whilst it is well established that pollutants in bushfire smoke, such as PM, can have a significant impact on human health, the possible impacts of bushfire smoke on cattle are not well described. As practically all cattle on pastures or in feedlots in Australia are outdoors, cattle may be exposed to significant quantities of smoke for extended periods during and following bushfire events.

There is a distinct lack of reports in the peer-reviewed literature relating to investigations into the potential consequences of chronic exposure of cattle to bushfire smoke. The objective of this review was to investigate evidence for likely effects on cattle from prolonged exposure to smoke generated from bushfires in Australia.

## 2. Materials and Methods

We initially used CAB Direct, PubMed, and Google Scholar to identify scientific papers by undertaking keyword searches, and scanning the abstracts to determine their relevance to the effects of chronic smoke exposure on cattle. For each set of keywords, the first 30 results were reviewed, and those deemed irrelevant to the research question were discarded. Initial search terms of “cattle smoke mortality” yielded no relevant articles through either CAB Direct or PubMed, but returned two relevant articles when using Google Scholar. Due to the limited research available, and wider searching capability of Google Scholar, the decision was made to continue investigating using Google Scholar alone. The search was expanded to include “cattle smoke effects”, and “fire smoke cattle”, which also brought little success. As expected due to the limited pool of research on explicitly cattle-related effects, searching more broadly for the constituents of bushfire smoke and the human impacts, using the search terms “bushfire smoke particulate matter PM”, was trialled with greater success, returning articles about bushfire smoke and its toxicity, and the human consequences of bushfire smoke exposure. Focus returned once more to cattle, now searching for evidence of ill-health as a result of other forms of air pollution, using the search terms “cattle air pollution”, and “cattle particulate matter PM” to scan for evidence of other sources of particulate matter affecting cattle health. Searching of other relevant government and educational resources using Google was also conducted, and relevant citations in the sourced literature were explored.

## 3. Results

A summary of the publications considered is found in Table 1.

### 3.1. Bushfire Pollution

Bushfire smoke comprises a combination of water vapour, end-products of combustion, and organic matter [10]. It is a significant contributor to air pollution levels in the affected area [32]. Many pollutants present in bushfire smoke are known to be toxic and detrimental to human and animal health, affecting on many body systems, particularly the respiratory system [3,33]. While there are hundreds of chemicals identified in bushfire smoke [3], some of the more significant components include toxic gases such as carbon monoxide (CO), carbon dioxide (CO_2_) and cyanide, oxides of nitrogen and pulmonary irritants such as acrolein, aldehydyes, ammonia (NH_3_), hydrogen bromide, hydrogen fluoride, isocyanide, nitrous gases and sulphur dioxide (SO_2_) [34]. Carbon monoxide is produced because of incomplete combustion of organic material [33], and binds haemoglobin, preventing oxygen transport and leading to hypoxia [33]. Cyanide gas can also be produced when plastics and asphalt are burned in bushfires [33], and can rapidly cause hypoxia. Nitrogen and sulphur-based compounds both cause respiratory irritation and can be present in bushfire smoke [18], but the nature of the fire determines whether nitrogen is reduced to compounds such as ammonia (in smouldering combustion), or oxidized to form nitrogen oxides (in flaming combustion) [18].

However, it is particulate matter generated in bushfires that is the air pollutant that increases to the greatest degree [7]. Particulate matter is a component of air pollution, that encompasses an array of aerosolized microscopic material, and in bushfires includes dust, ash, soot and metal oxides [10]. Particulate matter is considered the main agent in air pollution-related health effects [35], and so will be a major focus of this paper. Particulate matter can be classified as coarse, fine or ultrafine, depending on the diameter of the particles [36]. Coarse PM, often called PM_10_, refers to particulate matter with an aerodynamic equivalent diameter (AED) of between 10 µm and 2.5 µm; fine PM (PM_2.5_) has an AED of between 2.5 µm and 0.1 µm; and ultrafine PM (PM_0.1_) comprises particles with an AED less than 0.1 µm [37]. The characterisation of PM based on diameter is important, as it defines where the particles can settle in the lungs. All particles under 10 µm (PM_10_, PM_2.5_, PM_0.1_) can reach the lungs and cause issues, with fine and ultrafine components of PM of even greater concern given their ability to reach the alveoli, and the capability of ultrafine PM to enter the circulation [10,37]. With levels of PM_10_ in bushfires capable of reaching up to 10 times those of ambient levels [32], and on average 87% of related PM_10_ consisting of PM_2.5_ [38], there is a huge quantity of particulate matter present in bushfire smoke capable of negatively affecting on health. Although levels of PM_2.5_ are noted to be greatest at the source itself, and in the close surrounding area [39], the ability of particulate matter to travel large distances means that even humans and animals hundreds of kilometres away can be at risk [1]. Evidence suggests there are no safe exposure levels to several of the air pollutants found in bushfire smoke, including nitrogen dioxide, PM_10_ and PM_2.5_ [4]. It is important to note that much of the available previous research collected data only on PM_10_ measurements, which although includes all particulate matter less than 10 µm diameter, could not be separated into measurements of the various fractions and thus examining effects by PM fraction rarely was done. As technology has developed, more recent studies have been able to measure PM_2.5_ levels, and have determined that it is a major component of biomass smoke [40], with significantly increased levels during fire activity [40].

The National Environment Protection Council (NEPM) in Australia sets standards on ambient air quality, which determine acceptable levels of air toxics, such as particulate matter. Currently, the average daily limit on PM_10_ is 50 µg/m^3^, and 25 µg/m^3^ for PM_2.5_ [41]. The only situations in which concentrations higher than this are accepted are cases of an ‘exceptional event’, defined as a fire or dust occurrence affecting air quality at a particular location [41].

Another important consideration is the relative toxicity of bushfire smoke, and smoke or air pollution generated from industry. There is evidence that bushfire PM is substantially more toxic to the lungs than urban PM of the same concentration [42], indicating there are key differences between bushfire smoke and urban air pollution [18], and no safe level of bushfire smoke exposure [18].

Even between different bushfires, there can be differences in toxicity of smoke, as the chemical composition of bushfire smoke is influenced by the vegetation being burned [3]. It was found that burning eucalypts, which are dominant in large parts of the Australian landscape, results in the release of higher concentrations of PM_10_ than the burning of pine, acacia or cork oak [43]. One study looking at firefighter exposure on fire grounds also found that hotter, more intense bushfires were likely to lead to lower levels of toxic exposure for firefighters, compared to smouldering fires [44], evidence that both what is burned, and how it burns, has a significant impact on the smoke produced.

### 3.2. Effects of Bushfire Smoke on Cattle

Based on initial keyword search results, it soon became apparent that little research has been published specifically investigating bushfire smoke inhalation in cattle. One case study of a cow that had died per-acutely due to asphyxiation after a fire in a house adjacent to a shed [45]. There were several University-produced articles and a government department webpage that discussed the impacts of fires and smoke inhalation on livestock, but no peer-reviewed research.

These articles discussed the effects likely to be seen in animals directly affected by bushfires, including burns and smoke inhalation. They claimed the effects were similar in humans and livestock [46]. It was noted that PM in smoke could irritate the eyes and airways, and accumulate in the respiratory system, causing nasal discharge, bronchitis, lung inflammation and oedema and emphysema [45,46,47]. They reported that smoke may also worsen chronic cardiopulmonary diseases, such as congestive heart failure, chronic obstructive pulmonary disease (COPD), and asthma [46]. Signs such as increased respiratory effort, or persistent coughing, wheezing or nasal discharge might also be seen [46,48]. Reduced immune function and impaired airway clearance mechanisms due to PM may also render livestock such as cattle more susceptible to infection [46]. Severity of damage was correlated both with duration of smoke exposure, and concentration of smoke inhaled [47]. Damage might occur within minutes in thick smoke, or hours at lower concentrations [47]. After the smoke event, cattle should be watched closely for complications as airway damage heals over the next 4–6 weeks [46,47], although it is claimed that cattle that have not suffered burns typically experience no long-term effects [48]. Cattle may also suffer from productivity losses, as ash-covered pastures may be unpalatable [48].

Given the lack of peer-reviewed evidence on the effects of bushfire smoke on cattle, attention was focused on other PM sources that cattle were likely to be exposed to, such as air pollution and PM generated from livestock operations on farms, to determine if there was evidence that PM from other sources had a negative impact on cattle.

### 3.3. Effects of Air Pollution on Cattle

Exposure to air pollution produced because of livestock farming activity is well documented, particularly in farm workers and local communities, but studies examining direct effects on cattle are more limited. Concentrated animal feeding operations received much attention and were the focus of much of the research. The air in and around these farms is known to carry a high burden of pollutants [29], with close to 150 potentially toxic pollutants identified [31], including high levels of dust and particulate matter, as well as hydrogen sulphide (H_2_S), ammonia (NH_3_), volatile organic compounds and endotoxin [31]. For example, a study examining PM in Australian feedlots found that PM_10_ concentration reached as high as 792 µg/m^3^ [23], and had a 24-h average PM_10_ that approached or exceeded the Australian threshold of 50 µg/m^3^ twice in just 10 days of monitoring [23], indicating a possible cause of concern for workers and animals.

As previously noted, PM is considered the main agent responsible for air pollution-related health impacts [35], and as such, is a focus of much of the research. PM_2.5_ and ammonia can travel long distances and can impact on areas far from where they were produced [30]. Associations have been found between incidence of reduced human lung function and closeness to livestock farms, and increased ammonia levels have been correlated with increased airway obstruction [29].

Particulate matter generated in feedlots is also qualitatively different from that produced in bushfires. Particulate matter generated in feedlots and on farms comprises particles of soil dust, bedding, manure, feed, and microorganisms such as bacteria, viruses and fungi [31]. One study investigating sources of PM in feedlots found that manure was the largest contributor to PM_10_, accounting for 78% of the total PM_10_ load, while dust was responsible for 19% [25]. Particulate matter generated from soil dust is usually coarse material [31,46], which is of lesser concern than fine and ultrafine PM as it cannot penetrate as deeply in the lungs [37,46]. In contrast, Lee et al. [49] showed that over 50% of PM from animals in confinement, arising from manure and other material, was demonstrated to be much finer, at less than 3 µm [49]. However, the partially enclosed and ventilated confined environments where measurements were taken differ greatly from the predominantly outdoor systems seen in feedlots and farms in Australia, so it is likely that in these environments, PM from dust would account for a greater amount of PM. This is reflected in the work of Sweeten et al. [24] who found that 19–40% of total suspended particles (TSP) had a AED of less than 10 µm, and that, of the PM with an AED of less than 10 µm, PM_2.5_ made up only 5% [24].

Cambra-Lopez et al. [27] highlighted three ways PM might affect health. The first way is by inhalation which causes irritation of the respiratory tract and subsequently an increased risk of respiratory or cardiovascular disease [27]. Exposure to PM is related to oxidative stress, respiratory diseases, and increased levels of mortality [27]. One large study from the Netherlands found evidence of an increase in both acute and delayed effects of air pollution on dairy cow mortality, but only in the warmer months [8,21]. Over 87,000 dairy cow deaths were compared with daily PM_10_ concentrations, revealing a 10 µg/m^3^ increase in PM was associated with a 1.2–1.6% increase in same-day mortality, and a 3.2–5.1% increase in mortality over a 26-day cumulative estimate [8,21]. The authors reported that dairy cattle appeared more sensitive to air pollution than humans, and could potentially serve as sentinels for human health risks [21]. They noted that due to a small physiological gaseous exchange capacity, greater basal ventilatory activity, and greater anatomical compartmentalisation of the lung as compared with other mammals, cattle have a greater inherent susceptibility to pulmonary issues [8,50]. They argued that despite differences in cattle and humans, biochemical and physiological responses to air pollution were expected to be consistent [8]. Concentration of PM also appears to play a major role, with a study in the Netherlands finding no effect of PM_10_ on cattle mortality, however maximum PM_10_ levels found in the study were 85.8 µg/m^3^ [20]. In comparison, a study in pigs with ambient PM_10_ levels of 2000–3000 µg/m^3^ found increases in both pneumonia incidence and severity [51].

A second way PM can affect health is through irritation of the respiratory tract and lungs due to compounds present in or bound to PM that enhance its irritating effects, such as endotoxin, or compounds containing nitrogen or sulphur as are often found in intensive animal settings [31]. Polychlorinated hydrocarbons are capable of being bound to PM_2.5_ [52]. A study in Scotland found that areas exposed to incinerator air pollution containing polychlorinated hydrocarbons experienced greater levels of twin-births in the human population and on two small cattle farms in the vicinity [22]. This study was limited by its small sample size of cattle, however the shared association in larger numbers of human cases is notable.

A third way PM can affect health is through inhalation of PM carrying microorganisms, such as bacteria and fungi [27], which can be transported to the lungs attached to PM [29,31]. This appears to be a greater risk in the summer, as concentrations of airborne bacteria and fungal spores on farm were shown to be greater, reflecting seasonal variation [49].

Although much of the research on PM pollution from livestock operations focused on human health impacts, some evidence indicated that PM generated from on-farm activities could negatively affect the health of cattle. However, as there are significant differences in both the size and composition of PM generated from livestock operations compared with bushfire PM, drawing conclusions on the presumed effects of bushfire smoke based only on this evidence would be unwise. Attention was therefore directed at reviewing evidence regarding the impacts of bushfire smoke on humans.

### 3.4. Effects of Bushfire Smoke PM on Health

There are few reports about the effects of bushfire smoke PM on cattle. The findings of Cox et al. [8,21] indicates the potential suitability of using cattle as sentinels for human health and common pathophysiological patterns exhibited by air pollution and PM. It seems reasonable that the effects of bushfires smoke on humans and other animals could provide insight as indirect evidence to predict the likely effects of bushfire smoke on cattle.

### 3.5. Mortality

There were four studies identified which investigated effects on human mortality. These are described in Table 2.

Although studies investigating PM and mortality rates reported mixed results, three of the four studies found evidence of a positive association between PM levels and mortality rates. Of the studies, Johnston et al. [1], conducted the study which had the greatest applicability to the research question. The study was the longest of the studies investigating bushfire PM, and directly examined the increase in non-accidental mortality rates on days where PM_10_ exceeded the 99th percentile of its distribution due to fire activity. However, the study by Morgan et al. [11], conducted in the same locality, found no consistent association between bushfire PM_10_ and mortality rates. They reported that levels of urban PM_10_ levels were more associated with increased mortality. However, failing to find an association between bushfire PM and mortality rates may be due to the low number of daily deaths due to respiratory mortality, which limits the ability to find an effect even if one was present [11].

Importantly, there are limitations in extrapolating this data to cattle exposed to bushfire smoke in Australia. Background levels of urban PM in cities are likely to be much higher compared to extensively managed cattle at pasture. It is also likely there is a cumulative effect of PM, such that people chronically exposed to urban PM, which Morgan et al. [11], demonstrated was correlated with increased mortality, are at greater risk than cattle might be who usually enjoy much lower levels of PM. In the face of limited research, the idea that mortality effects might be lower in cattle exposed to bushfire PM is to be considered.

### 3.6. Respiratory and Cardiovascular Outcomes

There were seven studies identified which investigated effects on respiratory and/or cardiovascular outcome. These are described in Table 3.

The studies described in Table 3 that investigated the association between bushfire PM and respiratory outcomes found positive associations between increasing levels of bushfire PM and increased risk of respiratory morbidity. Associations between bushfire PM and cardiovascular effects were more varied. There was also mixed evidence as to whether bushfire PM was more likely to cause acute or chronic respiratory and cardiovascular morbidities.

From their review, Reisen & Brown [4] pointed out that healthy adults generally recovered quickly from short-term bushfire smoke exposure, and that adverse health effects were mainly seen in people who were more vulnerable, such as children, the elderly or pregnant women, or those with a pre-existing respiratory or cardiovascular issue. In the study by Haikerwal et al. [12], the exclusion of those <35 years old from the study is unlikely to paint a true picture of population level effects. It is reasonable to question whether a similar relationship might exist in cattle, wherein young or ill-thrifty animals, and high production animals such as those lactating or pregnant might also be more susceptible to deleterious effects on health and productivity.

An interesting association was that found by Johnston et al. [14], whose study from Darwin found that the positive associations for respiratory outcomes among the indigenous population was more than double that of the total population. The indigenous population also showed a positive association for ischaemic heart disease that was not evident in the total population. Whilst the authors attributed part of this discrepancy to the smaller sample size of indigenous people, it may also be effected by genetic factors, as genetics can have an influence on differences in susceptibility [28]. Applied to cattle, this might suggest that certain breeds might be more or less hardy or resilient to the effects of lingering smoke. This is speculative, as no research has been done.

The ability of humans to ‘self-report’ is another critical factor that limits the extent to which human studies can be extrapolated to cattle. Studies measuring respiratory and cardiovascular outcomes rely on humans first presenting themselves to emergency departments or to a physician.

There is also the risk of response bias, where public health advice on televisions or newspapers may influence perceived symptoms. For example, a phone survey investigating health effects in Albury, NSW, after a 38-day bushfire smoke event, reported that 70% of respondents claimed to have experienced at least one smoke-related health effect [15]. Whether 70% of the population were actually affected or whether they were influenced by public health announcements can be challenging to assess, and may lead to an overestimation of true cases.

In contrast, morbidity in cattle is likely to be less noticed, and the effects of bushfire smoke may remain more undetected. Prey animals such as cattle will naturally hide signs of discomfort, and it may only be an attentive farmer that notices affected cattle. Frequency of monitoring, and size of herd would have a large impact on detection of health impacts. For instance, effects may be seen less in beef herds, compared to dairy herds where cattle are observed by the farmer twice per day. In herds of a few hundred cattle, it would be easy to miss a few cases of nasal discharge, or watery eyes, or coughing. Likewise, due to the reduced strength of smaller populations to detect an effect, the death of one or two animals during a period of smoke haze may be dismissed.

### 3.7. Animal Studies

A 2018 review by Reczynska et al. [55], examining animal models of smoke inhalation injury reported no studies on smoke inhalation modelling performed on cattle, so other animal studies on smoke inhalation injury were examined. A 1982 animal study investigated the effects of smoke inhalation on lung tissue in rabbits [56], after they were exposed to cool smoke for between 25 and 45 min. Six hours after exposure, there was a significant inflammatory response throughout the pulmonary tissue, and by 24 h sloughing and necrosis of epithelial cells had resulted in formation of a pseudomembranous exudate, and oedema was present. By 72 h post-injury, a non-ciliated epithelium was forming and oedema and inflammatory response had decreased. [56] Lack of a ciliated epithelium means that airway clearance depends then on the cough mechanism. [56] The study did not determine whether the ciliated epithelium would return. A second study on smoke inhalation in anaesthetized sheep found similar results, but epithelial necrosis and sloughing were evident only 15 min after exposure. [57] They found that extent of injury was directly related to smoke exposure dose. By 72 h, bacterial infection was present in severely affected sheep. Of the sheep that received a high dose of smoke and died, all did so due to severe airway obstruction and subsequent hypoxia. [57] They found that in sheep that survived mild or moderate smoke exposure, bronchopneumonia resolved and respiratory epithelium healed and normal cilia was present within 2 and 4 weeks, respectively.

While these studies detail the consequences of smoke inhalation, they are limited in answering the question of how bushfire smoke is likely to affect cattle at pasture. In both of these studies, exposure was acute and enclosed, in a small exposure chamber or by endotracheal intubation, so direct comparison between this and open-air exposure in cattle is difficult. This might suggest that the human studies, which examined smoke inhalation within ambient air, are more comparable. However, these studies do highlight how smoke inhalation can have significant effects on the airways in animals, and importantly note that healing can occur if smoke exposure is not severe. However, defining what constitutes ‘severe’ smoke exposure is difficult, as neither study measured concentration of particulate matter in the smoke delivered, and different animals exhibited variations in response to equivalent doses.

### 3.8. Reducing the Risks

Minimising risks where possible is prudent, not only to protect the animals’ health, but also to reduce any potential consequences smoke may have on productivity. During periods of smoke haze, increased monitoring of water sources for quality and contamination may be needed, as settled ash may reduce willingness of stock to drink. Fallen ash on pasture may also make pasture unpalatable for stock, and farmers may have to increase bail feeding [48]. Reducing physical stressors on cattle exposed to bushfire smoke would also appear to be important. Moving smoke-exposed cattle only if necessary, and in a low-stress way may help to reduce exposure. During physical activity, pulmonary ventilation increases and more pollutants are inhaled, and there is an increase in mouth-breathing, allowing more air to avoid the normal nasal filtration [44]. Airflow velocity is also higher during increased physical activity, which facilitates the transport of PM deeper into the lungs, increasing the risk of respiratory effects [44].

## 4. Conclusions

Whilst there is evidence that particulate matter from bushfire smoke has a measurable impact on both human mortality and cardiorespiratory morbidities, there are few reports describing the impact of chronic bushfire smoke exposure in cattle.

We conclude that cattle do not appear likely to be severely impacted by chronic smoke haze exposure, as evidenced by the lack of reports.

We hypothesise this may be because cattle do not tend to suffer from the co-morbidities that, in the human population, seem to be made worse by smoke and pollution.

Further, small changes to background mortality rates or transient morbidity—even if they occurred across many smaller herds—may go unreported.

Whilst it is clear in humans that constituents in fire smoke cause trauma to the airways when inhaled in high doses or for significant exposure periods, the ability to heal and regain function in a matter of weeks suggests that effects, if present in cattle, are likely to be transient. However, the lack of formal research into the effect of bushfire smoke on cattle makes this conclusion speculative, and further investigation is warranted.

## Figures and Tables

**Table 1:** Results of a literature search to identify the effect of bushfire smoke on cattle identified by keywords, 1–3 April 2020.

Keywords	Title	Summary
Cattle smoke mortality	Ambient air pollution-related mortality in dairy cattle: does it corroborate human findings? (Cox et al., 2016) [8]	Investigated whether the short-term association between air pollution and mortality in humans could be corroborated in an animal population.
	Cause-specific mortality and the extended effects of particulate pollution and temperature exposure. (Goodman, Dockery & Clancy, 2004) [9]	Investigated associations of particulate pollution (black smoke) and temperature with age-standardized daily mortality rates over 17 years in Dublin, Ireland.
Cattle smoke effects	N/A	
Fire smoke cattle	Where there’s fire, there’s smoke: air quality & prescribed burning in Florida. (Monroe, Watts & Kobziar, 1999) [10]	Background information on air quality, the effects of smoke on human health and safety, regulations concerning the use of prescribed fires and the smoke produced by them.
Bushfire smoke particulate matter PM	The effects of bushfire smoke on respiratory health. (Dennekamp & Abramson, 2011) [7]	Examined the effects of bushfire smoke on human respiratory health.
	Effects of bushfire smoke on daily mortality and hospital admissions in Sydney, Australia. (Morgan et al., 2010) [11]	Investigated associations of daily mortality and hospital admissions with bushfire-derived particulates, compared with particulates from urban sources in Sydney, Australia from 1994 to 2002.
	Impact of fine particulate matter (PM_2.5_) exposure during wildfires on cardiovascular health outcomes. (Haikerwal et al., 2015) [12]	Examined the associations of out-of-hospital cardiac arrests, ischaemic heart disease, acute myocardial infarction, and angina from hospital admissions and emergency department attendance, with PM_2.5_ concentrations during the 2006–2007 bushfires in Victoria, Australia.
	Three measures of forest fire smoke exposure and their associations with respiratory and cardiovascular health outcomes in a population-based cohort. (Henderson, Brauer, MacNab & Kennedy, 2011) [13]	Examined the associations between respiratory and cardiovascular physician visits and hospital admissions, and three measures of smoke exposure over a 92-day study period (July–September 2003).
	Extreme air pollution events from bushfires and dust storms and their association with mortality in Sydney, Australia 1994–2007. (Johnston et al., 2011) [1]	Retrospectively assessed the mortality associated with extreme air pollution events due to bushfire smoke and dust in Sydney from January 1994 to June 2007.
	Ambient biomass smoke and cardio-respiratory hospital admissions in Darwin, Australia. (Johnston, Bailie, Pilotto & Hanigan, 2007) [14]	Examined the relationship between atmospheric particle loadings <10 microns in diameter (PM_10_), and emergency hospital admissions for cardio-respiratory conditions over the three fire seasons of 2000, 2004 and 2005.
	An extreme bushfire smoke pollution event: health impacts and public health challenges. (Kolbe & Gilchrist, 2009) [15]	Described a bushfire smoke event, the role of public health during the event and a survey conducted to determine the health impacts of smoke and the effectiveness of public health advisories.
	Risk of respiratory & cardiovascular hospitalisation with exposure to bushfire particulates: new evidence from Darwin, Australia. (Crabbe, 2012) [16]	Analysed data from Darwin, Australia in the 1990s, to investigate the relationship between bushfire smoke and hospital admissions.
	Long-range fine particulate matter from the 2002 Quebec forest fires and daily mortality in Greater Boston and New York City (Zu et al., 2016) [17]	Examined the association between PM_2.5_ and mortality in Greater Boston and New York City during and after forest fires in Quebec, Canada blanketed the US East Coast in smoke.
	Bushfire smoke: an exemplar of coupled human and natural systems (Johnston & Bowman, 2014) [18]	A review of the impacts of wildfire smoke on human health.
	Implications for community health from exposure to bushfire air toxics (Reisen & Brown, 2006) [4]	A review focusing on the air pollution generated by bushfires and the impacts on people’s health.
	Particulate air pollution from bushfires: human exposure and possible health effects (Karthikeyan, Balasubramanian & Iouri, 2006) [19]	Investigation of the trace metal characteristics of airborne PM_2.5_ collected in Singapore from February-March 2005.
	In vitro assessment of the toxicity of bushfire emissions: A review (Dong et al., 2017) [3]	A review focusing on the toxicity of bushfire smoke using results obtained from in vitro studies.
Cattle air pollution	Short-term effects of air pollution and temperature on cattle mortality in the Netherlands (Egberts, van Schaik, Brunekeef & Hoek, 2019) [20]	Investigation of the effects of daily variations in air pollution levels and ambient air temperature on cattle mortality between 2012 and 2017.
	Cattle mortality as a sentinel for the effects of ambient air pollution on human health (Cox et al., 2015) [21]	Investigation of the association between ambient air pollution and mortality in dairy cows from 2006–2009.
	Twinning in human populations and in cattle exposed to air pollution from incinerators (Lloyd, Lloyd, Williams & Lawson, 1988) [22]	Investigated the hypothesis that an association between twinning and chemical pollution would be found among cattle and people in areas near incinerators in Bonnybridge, Scotland.
Cattle particulate matter PM (focusing on articles that explore health impacts)	Coarse particulate matter emissions from cattle feedlots in Australia (McGinn et al., 2010) [23]	Measured PM_10_ concentrations and emissions at two cattle feedlots in Australia over several days to evaluate a technique to calculate short-term PM_10_ emissions from the feedlot.
	Particle size distribution of cattle feedlot dust emission (Sweeten, Parnell, Shaw & Auvermann, 1998) [24]	Compared field data from both total suspended solids (TSP) and PM_10_ samplers at cattle feedlots to compare particle size distribution of PM.
	Utilising single particle Raman microscopy as a non-destructive method to identify sources of PM_10_ from cattle feedlot operations (Huang et al., 2013) [25]	Aimed to develop a non-destructive method to determine the source profile of PM_10_ particles emitted from the cattle feedlot.
	Dust emissions in cattle feedlots (Sweeten, Parnell, Etheredge & Osborne, 1988) [26]	Determined the concentration of dust emitted from feedlot surfaces, to determine the particulate size distribution of feedlot dust, and to determine if dust emissions were correlated with surface manure moisture content.
Livestock particulate matter PM health	Airborne particulate matter from livestock production systems: a review of an air pollution problem (Cambra-Lopez et al., 2010) [27]	Summarized the major problems associated with PM in livestock production systems.
	Airborne particulate matter and human health: a review (Davidson, Phalen & Solomon, 2005) [28]	A summary of the impacts of particulate matter on human health, including sources of PM, places of exposure, susceptibility, and reducing exposure.
	Air pollution from livestock farms is associated with airway obstruction in neighbouring residents (Borlee et al., 2017) [29]	Investigated associations between spatial and temporal variation in pollutant emissions from livestock farms and lung function in a rural, non-farming population in the Netherlands.
	Impacts of intensive livestock production on human health in densely populated regions (Smit & Heederik, 2017) [30]	Highlighted the respiratory health effects of non-infectious air pollutant emissions from livestock farms.
	Worker health and safety in concentrated animal feeding operations (Mitloehner & Calvo, 2008) [31]	A review that focused on accidental injury and air pollution as areas of major concern to the health and safety of farm workers.

**Table 2:** Summary of studies investigating PM and human mortality using search terms “Cattle smoke mortality” or “Bushfire smoke particulate matter PM” in Google Scholar.

Summary	Exposure	Outcome	Comments
Johnston et al. [1] investigated the correlation between bushfire smoke pollution and mortality rates in Sydney over a 13.5 year period, using results from over 28,400 deaths	46 days classed as smoke events, in which PM_10_ levels were greater than 47.3 µg/m^3^ (equivalent to the 99th percentile of its distribution)	5% increase in non-accidental mortality	No information on more specific causes of death
Goodman, Dockery & Clancy [9] investigated the correlation between increased concentrations of black smoke from coal burning, and mortality rates over 17 years in Dublin, Ireland, using data from over 80,000 deaths	Results calculated on a per-10 µg/m^3^ increase in black smoke basis	Increases in mortality, both acutely (0.4%) and delayed up to 40 days (1.1%) Respiratory causes of mortality also increased, both acute (0.9%) and delayed (3.6%)	Explored effects of smoke from coal burning rather than bushfires Used black smoke (BS) as a measure, rather than PM
Zu et al. [17] correlated daily mortality rates in Boston and New York with elevated PM_2.5_ levels from bushfires over a four week period	PM_2.5_ levels	No increases in mortality	This might be explained by variation in PM chemical compositions, with health effects determined not by any single chemical but as a result of a combination of chemical constituents, making some PM more dangerous than others. [28]
Morgan et al. [11] conducted a study in Sydney over 8 years investigating rates of mortality and hospital admissions in relation to fire-associated PM and urban PM	Data on daily mortality was compared to PM_10_ concentrations; 32 days with PM_10_ levels above the 99th percentile were associated with fires, and the rest were associated with PM_10_ generated from urban pollution	No consistent association was found between bushfire PM_10_ and mortality rates, but urban PM_10_ was associated with cardiovascular and respiratory mortality	

**Table 3:** Summary of studies investigating PM and respiratory and/or cardiovascular outcomes *.

Summary	Exposure	Respiratory Outcomes	Cardiovascular Outcomes	Comments
Morgan et al. [11] conducted a study in Sydney over 8 years investigating rates of mortality and hospital admissions in relation to fire-associated PM and urban PM	32 days where PM_10_ levels were above the 99th percentile were associated with fires. Admission results were calculated on a per-10 µg/m^3^ increase in PM_10_	1.24% increase in admissions	No association	Although authors accounted for lag effects up to 7 days post-exposure, they found the effects of bushfire PM on respiratory admissions to be more acute, with admissions within the first 3 days after the smoke event
Reid et al. [39] examined associations between respiratory hospital admissions and bushfire PM_2.5_ during the 2008 bushfires in California	Admission results calculated on a per-10 µg/m^3^ increase in PM_2.5_	4% increase for asthma admissions 14% increase for COPD admissions	Not reported	This is likely due to the high level of PM_2.5_ during the fires, which had an average concentration nearly three times greater than levels before the fires
Tham et al. [53] examined associations between higher daily PM_10_ levels from bushfire smoke in Victoria and respiratory outcomes (admissions and emergency department (ED) visits)	Measured 24-h average daily PM_10_ concentrations	Strong association with ED visits (1.8%) Weak association with hospital admissions (0.3%)	Not reported	No significant associations were found between PM_10_ and respiratory outcomes in Gippsland—noted this may be due to smaller population size
Henderson et al. [13] conducted a study in Canada, examining measures of smoke exposure and associations with respiratory and cardiovascular health outcomes	Associations measured on a per-10 µg/m^3^ increase in PM_10_	5% increase for respiratory admissions 6% increase in asthma-specific doctor’s visits	No association	
Johnston et al. [14] conducted a 3 year study in Darwin, Australia, investigating the link between bushfire smoke & cardio-respiratory hospital outcomes (2466 admissions)	Associations measured on a per-10 µg/m^3^ increase in PM_10_	8% increase in all respiratory admissions 13% increase for asthma admissions 20% increase for COPD admissions NB: these associations more than doubled in the indigenous community	No association NB: indigenous people showed a positive association for ischaemic heart disease	The increased positive association witnessed in indigenous people may suggest a genetic component of increased susceptibility to cardio-respiratory diseases. However, noted this large effect may in part be due to the smaller sample size of indigenous people, who made up only 23% of cases. One of the unique aspects of this study was the significant lack of industrial air pollution. Noted that 95% of air pollution in Darwin is caused by fires in the surrounding scrub.
Delfino et al. [54] studied hospital admissions and the California wildfires of 2003 and found significant associations between PM_2.5_ levels and health outcomes	Associations measured on a per-10 µg/m^3^ increase in PM_2.5_	9.6% increase for acute bronchitis 6.9% increase for COPD (ages 20–64) 6.4% increase in pneumonia (ages 5–18) Asthma admissions also increased (10.1% for ages 65–99, 8.3% for ages 0–4, 4.1% for ages 20–64)	Cardiovascular & congestive heart failure admissions increased after the fires	The elderly, and young children were most adversely affected by PM_2.5_. Authors noted that chronic exposure over several days could lead to greater levels of systemic inflammation, as cardiovascular admissions as well as acute bronchitis and pneumonia admissions increased after the fires, suggesting the effects of smoke inhalation may be delayed for weeks to months. It also might suggest that prolonged effects of bushfire PM increases susceptibility to later respiratory infections, due to reduced immune or respiratory clearance functions
Haikerwal et al. [12] examined cardiovascular health effects in relation to PM_2.5_ concentrations during the 2006–2007 bushfires in Victoria, Australia.	Associations measured on a per-9 µg/m^3^ increase in PM_2.5_ over a 2-day exposure period During the fires, maximum daily PM_2.5_ concentration reached as high as 100 µg/m^3^, well above the daily NEPA standard of 25 µg/m^3^		6.98% increase in out-of-hospital cardiac arrests 2.07% increase in ischaemic heart disease-related ED presentations	Authors identified that PM_2.5_ from smoke may be a contributing factor for acute coronary injuries during bushfires. The majority of the patients in the study were >65 years old, and the study excluded those <35 years. Authors noted that sustained exposure to bushfire smoke could lead to an inflammatory cascade in the body, which could later progress to heart disease and arrhythmias.

* Studies retrieved using keywords “Bushfire smoke particulate matter PM” and through following references found by that search.

## Data Availability

Not applicable.
